# Base excess and lactate as prognostic indicators for patients admitted to intensive care - 15 years later

**DOI:** 10.1186/2197-425X-3-S1-A341

**Published:** 2015-10-01

**Authors:** M Santi, G Mcanulty, M Grounds

**Affiliations:** General Intensive Care, St George's Hospital, London, United Kingdom

## Introduction

Identifying patients who may deteriorate on the ward and need admission to an intensive care unit (ICU) is imprecise. Numerous variables, alone or in combination, have been used to identify patients who may benefit from ICU admission. A predictive test should be accurate, reliable, easy to measure and cheap. [1]

15 years ago an observational study from our unit showed that both base excess (BE) and lactate, or combination of both, can be used to predict outcome in patients admitted to ITU [2]. We replicated this study to find out if BE and lactate are still useful prognostic indicators for patients admitted to ITU.

## Methods

As in the 2001 study we examined the records of 148 unselected, consecutive general medical and surgical admissions to St George's Hospital General ITU in September 2013. We recorded blood BE and lactate levels at admission and at 24 hours and calculated corresponding Sequential Organ Failure Assessment (SOFA) scores. The data was taken from the patients' ITU charts and our computer laboratory result system EPR. We compared this data to that presented in 2001. The chi square test was used to determine the statistical difference between groups.

## Results

44 patients were admitted with acute medical problems, 68 after elective surgery and 36 after emergency surgery (compared with 70, 53 and 25 respectively in reported in 2001). As in 2001 we divided the patients into three groups: those with admission serum lactate ≥ 1.5 or < 1.5 mmol/l, those with serum BE > -4.0 or ≤ -4.0 mmol/l and those with admission BE ≤ -4.0 mmol/l and a lactate ≥1.5mmol/l. Calculated admission SOFA scores were not significantly different between and of the 2001 and 2014 groups.

Patients with a deranged admission lactate had a mortality of 10%, versus 4.4% in the normal lactate group. If the lactate failed to clear at 24 hours, mortality was 32% versus 2% if it had cleared. Similarly, patients admitted with a BE ≤ -4.0 mmol/l had a mortality of 15% compared to 4% in patients with a BE >-4mmol/l. If BE was still ≤ -2.5 mmol/l at 24 hours, mortality was 25% versus 0% if the base deficit had improved. A combination of deranged lactate and BE ≤ -4.0 mmol/l showed a mortality of 17% and 33% if it had failed to clear at 24 hours. (Table 1)

**Figure 1:**
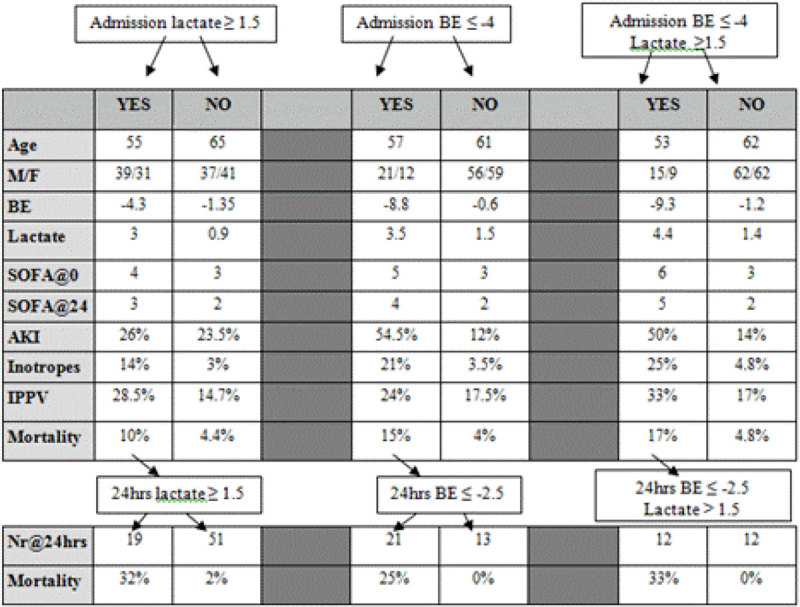
**Overview of lactate and base excess.**

## Conclusions

Base excess and lactate are still indicators of severity of disease and can be used to monitor treatment and response.
